# Phosphoprotein phosphatase-2A docks to Dishevelled and counterregulates Wnt3a/β-catenin signaling

**DOI:** 10.1186/1750-2187-2-12

**Published:** 2007-10-25

**Authors:** Noriko Yokoyama, Craig C Malbon

**Affiliations:** 1 Department of Pharmacology, Health Sciences Center, State University of New York at Stony Brook, Stony Brook, NY 11794-8651, USA

## Abstract

**Background:**

Wnt3a stimulates cellular trafficking of key signaling elements (*e.g*., Axin, Dishevelled-2, β-catenin, and glycogen synthase kinase-3β) and primitive endoderm formation in mouse F9 embryonic teratocarcinoma cells.

**Results:**

The role of phosphoprotein phosphatase-2A in signaling of the Wnt/β-catenin/Lef-Tcf-sensitive gene activation pathway was investigated. Wnt3a action attenuates phosphoprotein phosphatase-2A activity and stimulates the Lef/Tcf-sensitive gene transcription. Inhibiting phosphoprotein phosphatase-2A by okadaic acid, by treatment with siRNA (targeting the C-subunit of the enzyme), or by expression of SV40 small *t *antigen mimics Wnt3a action, increasing the cellular abundance of Axin and phospho-glycogen synthase kinase-3β as well as the trafficking of signaling elements in the Wnt/β-catenin pathway. Although mimicking effects of Wnt3a on the cellular abundance and trafficking of key signaling elements in the Wnt canonical pathway, suppression of phosphatase-2A alone did not provoke activation of the Lef/Tcf-sensitive transcriptional response, but did potentiate its activation by Wnt3a. Phosphoprotein phosphatase-2A and the scaffold phosphoprotein Dishevelled-2 display similarities in cellular trafficking in response to either Wnt3a or suppression of the phosphatase. A docking site for phosphoprotein phosphatase-2A in the DEP domain of Dishevelled-2 was identified.

**Conclusion:**

In current study, we showed new roles of phosphoprotein phosphatase-2A in Wnt/β-catenin signaling pathway: effect on protein expression, effect on protein trafficking, retention of molecules in subcellular compartments, and regulation of enzymatic activity of several key players. Docking of phosphoprotein phosphatase-2A by Dishevelled-2 suppresses phosphatase activity and explains in part the central role of this phosphatase in the counterregulation of the Wnt/β-catenin signaling pathway.

## Background

Phosphoprotein phosphatase-2A (PP2A), a phosphoserine/phosphothreonine-specific phosphatase, is expressed ubiquitously in cells and has been implicated as functioning in cell signaling, regulating the activity of protein kinases, protein phosphatases, and/or their substrates [1-3] as well as functioning in cellular metabolism, DNA replication, transcription, RNA splicing, translation, cell-cycle progression, and development [4,5]. PP2A is a multi-subunit enzyme, composed of three subunits termed conserved catalytic (C), structural (A) and a variable regulatory (B) [2,3] that form a stable complex with various protein kinases and regulate their activities [2,3].

Activation of the Wnt/β-catenin "canonical" pathway provokes inactivation of GSK3β, stabilization and accumulation of nuclear β-catenin, and activation of β-catenin-sensitive Lef/Tcf-dependent gene transcription. PP2A has been implicated in Wnt signaling at several levels, interacting with the Axin-based β-catenin destruction complex [6] as well as demonstrating a marked influence on Wnt signaling in *Xenopus *downstream of β-catenin [7]. PP2A can catalyze the dephosphorylation of Axin and of the product of the *adenomatous polyposis coli *gene (APC) [8,9] as well as of phospho-GSK3β [10]. PP2A regulatory subunit B56 also interacts with the product of the APC gene, influencing β-catenin stability [11,12]. Furthermore, expression of the PP2A family member B56ε in *Xenopus *is essential for early development [13]. In the current study, we investigate roles of PP2A, specifically in regulating the abundance and trafficking of key signaling elements of the Wnt/β-catenin "canonical" pathway [14-18]. The results highlight the role of PP2A action in regulating the abundance, trafficking, and function of several key signaling elements in the Wnt/β-catenin pathway. Inhibition of PP2A action is Wnt3a-mimetic with respect to regulating the cellular abundance and trafficking of signaling element as well as potentiating the Wnt3a-stimulated transcriptional response. In addition, we identify a new docking site for PP2A, localized to the DEP domain of Dishevelled-2 (Dvl2).

## Results

### Okadaic acid potentiates Wnt/β-catenin signaling

**Figure 1:**
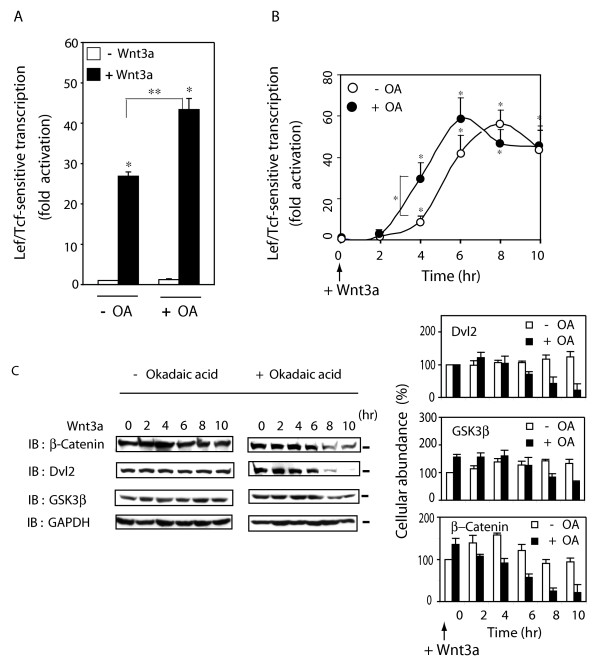
**Suppression of PP2A potentiates the Wnt/β-catenin signaling**. *Panel A*, OA enhanced the Lef/Tcf-sensitive transcription activity in response to Wnt3a. F9 cells expressing Rfz1 were treated with Wnt3a for 8 hr in the absence or presence of OA, added 60 min prior to stimulation with Wnt3a. Cells were lysed and the Lef/Tcf-sensitive gene transcription was assayed in samples of cell lysates using the M50 luciferase gene reporter. The results showed mean values ± S.E. that were obtained from five separate experiments. Statistical significance is noted (*, *p *< 0.001; **, *p *< 0.05). *Panel B*, OA enhanced the time-course of activation of Lef/Tcf-sensitive transcription in response to stimulation by Wnt3a. F9 cells expressing Rfz1 were treated without (open circle) or with (closed circle) OA and Wnt3a added for a 10 hr stimulation. Cells were harvested at indicated time points and disrupted. The Lef/Tcf-sensitive gene transcription was assayed. The results shown are mean values ± S.E. obtained from four separate experiments. Statistical significance is denoted (*, for *p *< 0.005). *Panel C*, effects of OA on the cellular abundance of Wnt/β-catenin signaling elements in response to long-term (0–10 hr) stimulation by Wnt3a. The cellular content of β-catenin, Dvl2, GSK3β, and GAPDH (as a control) were established in F9 cells expressing Rfz1 and stimulated with Wnt3a, in the absence or presence of OA, for 0–10 hr. The results shown are mean values ± S.E. obtained from four separate experiments. Representative blots are displayed.

The mouse F9 teratocarcinoma cells (F9 cell) were transfected with an expression vector harboring rat Frizzled-1 (Rfz1) [19]. In previous studies, we demonstrated that these cells display both cytoplasmic and nuclear accumulation of β-catenin, activation of Lef/Tcf-sensitive gene transcription, and primitive endoderm formation in response to Wnt3a [19-22], providing an ideal model for the current study. The ability of PP2A to dephosphorylate several members of the Axin-based degradation complex *in vitro *and the central role of phosphorylation in the canonical Wnt/β-catenin pathway provoked our interest in probing its role in the trafficking of these signaling molecules. We first made use of okadaic acid (OA), a chemical inhibitor of serine/threonine phosphoprotein phosphatases (PP1 and PP2A), and probed its effects on Lef/Tcf-sensitive gene transcription in response to Wnt3a (figs. 1A, B). Basal activity of Lef/Tcf-sensitive gene transcription was unaffected by OA. Cells were treated with OA (30 nM) for 1 hr and then stimulated with purified Wnt3a (fig. 1A). OA elevated the Lef/Tcf-sensitive transcriptional response to Wnt3a from 25-fold to 45-fold, although not altering significantly the basal transcriptional response (fig. 1A). The ability of Wnt3a to stimulate Lef/Tcf-sensitive gene transcription in the absence or presence of OA was probed over time (fig. 1B). OA enhanced temporally the activation of Lef/Tcf-sensitive transcription in response to Wnt3a, half-maximal activation at 4 hr post Wnt3a; the amplitude of the response was not altered (fig. 1B).

The cellular abundance of β-catenin, Dvl2 and GSK3β in response to Wnt3a stimulation were investigated in the OA-treated cells (fig. 1C). OA potentiated the Lef/Tcf-sensitive transcriptional response to Wnt3a, while increasing the cellular content of GSK3β and β-catenin, but not Dvl2 (fig. 1C). Stimulating the OA-treated cells with Wnt3a for up to 4 hr had little influence on the cellular content of Dvl2, GSK3β, or β-catenin, although provoking a longer-term (6 to 10 hr) decline in their cellular abundance. For example, suppression of PP2A activity and stimulation by Wnt3a provoked a sharp decline (~80%) in Dvl2 by 8- to 10-hr (fig. 1C). PP2A activity appears to be critical to maintaining decreased phosphorylation of all three molecules, which increases their stability and cellular content over the long term.

### Suppressing PP2A action either by siRNA or by expression of SV40 small t antigen potentiates the Lef/Tcf-sensitive transcriptional response to Wnt3a, mimicking OA

**Figure 2:**
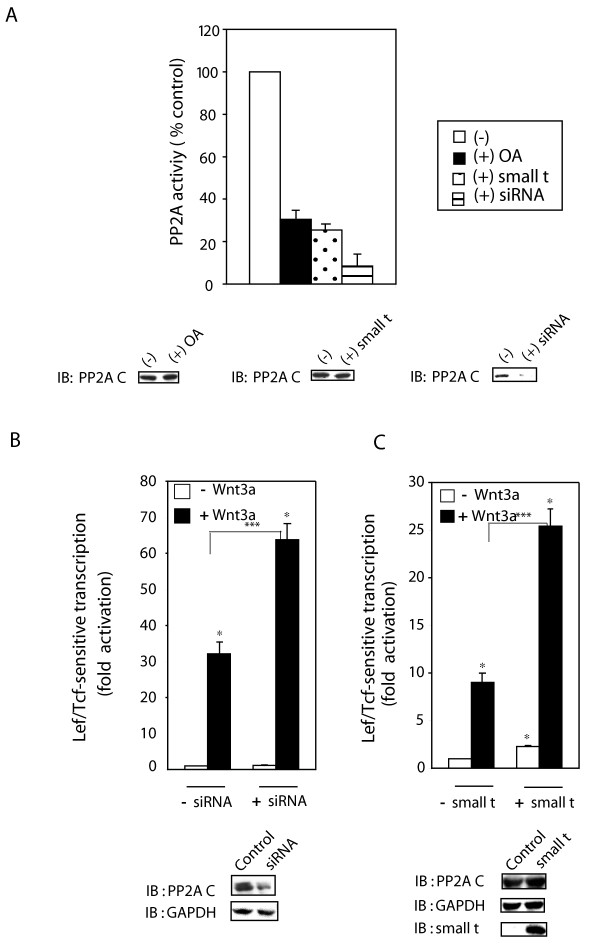
**Suppression of PP2A activity by siRNA or small t antigen enhances Lef/Tcf-sensitive transcription**. *Panel A*, Rfz1-expressing F9 cells were treated with OA for 1 hr or siRNA targeting PP2A C subunit for 48 hr, or co-expression of small *t *antigen for 48 hr. Cell lysates were applied to a small Sephadex-G50 column and PP2A activity assay was carried out using pNPP as a substrate. The results are shown as mean values ± S.E. from 8–10 independent experiments. Abundance of PP2A C subunit was determined by Western immunoblotting with anti-PP2A C subunit antibody. *Panel B*, cells were treated with siRNAs targeting the PP2A C-subunit for one day before co-transfection of the cells with Rfz1 and Super8xpTOPFlash plasmids. Cells were stimulated with or without Wnt 3a for 8 hr. The luciferase gene reporter was assayed and is displayed relative to the unstimulated cells (set to "1"). The results showed mean values ± S.E., obtained from five separate experiments. Statistical significance is indicated (*, *p *< 0.001; ***, *p *< 0.005). Cell extracts also were analyzed for abundance of PP2A C-subunit by immunoblotting. Immunoblots were stained with anti-GAPDH antibodies to establish loading equivalence.*Panel C*, activation of Lcf/Tcf-sensitive transcription was assayed in F9 cells co-transfected for one-day with Rfz1, Super8xTOPFlash (M50) and small *t *antigen then stimulated without and with purified Wnt3a for 8 hr. The luciferase gene reporter was assayed and the transcriptional response displayed relative to the unstimulated cells (set to "1"). Cell lysates were analyzed by immunoblotting, blots stained with anti-PP2A C-subunit, anti-GAPDH, or anti-small *t *antigen antibodies. The results shown are mean values ± S.E. from 5 independent experiments.

Two strategies in addition to OA treatment were tested to probe further the role of PP2A in Wnt3a stimulation of the Lef/Tcf-sensitive transcription. We compared the effects siRNA targeting the expression of the conserved catalytic or "C-"subunit of PP2A, the expression of SV40 small *t *antigen, inhibitor of PP2A, and the effects of OA (40 nM) treatment on PP2A activity (fig. 2A). OA reduces PP2A activity in this assay by >70%. Expression of small *t *antigen has been shown to inhibit PP2A activity [23,24] and transfection of F9 cells with an expression vector harboring the small *t *antigen reduced PP2A activity by ~70%. Treating the cells with siRNAs targeting the expression of PP2A C-subunit effectively suppresses C-subunit expression, reducing PP2A activity by ~90%.

If PP2A is a primary target of OA that influences the Wnt/β-catenin pathway, then either suppression of the PP2A C-subunit by siRNA or expression of SV40 small *t *antigen would be predicted to mimic the effect of OA on the Wnt3a-stimulated Lef/Tcf-sensitive transcription. Like OA treatment (figs. 1A, B), suppression of PP2A C-subunit expression by siRNA (fig. 2B, inset immunoblot of C-subunit expression with GAPDH expression employed as a control) was found to potentiate the Lef/Tcf-sensitive transcriptional response to stimulation by Wnt3a (fig. 2B). Basal Lef/Tcf-sensitive transcription remains essentially unchanged in these siRNA-treated cells. siRNA-induced suppression of PP2A C-subunit likewise enhanced (*i.e*., reduced) the time to reach half-maximal activation of Lef/Tcf-sensitive transcription by Wnt3a to 4 hr (results not shown), much like OA (fig. 1A).

Expression of small *t *antigen, like OA and suppression of PP2A C-subunit, also potentiated the Lef/Tcf-sensitive transcriptional response to Wnt3a (fig. 2C). The cellular levels of GAPDH and of PP2A were unaffected by expression of small *t *antigen. Small *t *antigen expression, in contrast to OA or siRNA targeting C-subunit, increased Lef/Tcf-sensitive transcription in the absence of Wnt3a by ~2.5-fold. Although each strategy (*i.e*., treatment with a chemical inhibitor versus siRNA treatment versus expression of small *t *antigen) displays some unique effect on the Super8XTOPFlash transcriptional readout itself, suppression of PP2A activity either by OA, or by siRNA, or by expression of small *t *antigen clearly potentiated the Lef/Tcf-dependent transcriptional response to Wnt3a (figs. 1, 2). Taken together, these three independent strategies demonstrate that PP2A action negatively modulates the Wnt3a/β-catenin canonical pathway.

### PP2A action suppresses the cellular content of Axin, β-catenin, and phospho-GSK3β

**Figure 3:**
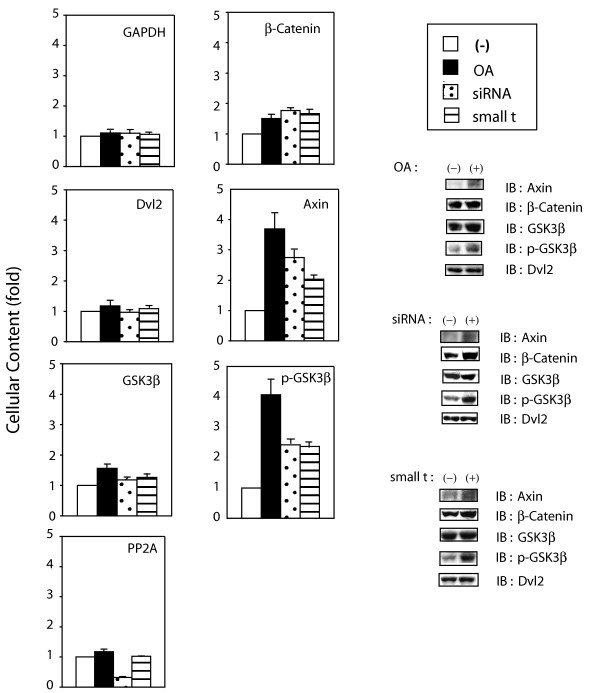
**Suppression of PP2A activity alters cellular abundant of Wnt/β-catenin signaling elements**. F9 cells expressing Rfz1 were either pretreated with OA for 1 hr or treated with siRNA targeting PP2A C- subunit for 48 hr, or transfected to express SV40 small *t *antigen for 48 hr. Cells were washed with PBS twice and lysed. Cell lysates were subjected to SDS-PAGE and analyzed by immunoblotting, blots stained with anti-Axin, anti-β-catenin, anti-Dvl2, anti-GSK3β, anti-p-Ser (9)-GSK3β, anti-PP2A C or anti-GAPDH antibody. The relative amounts of the proteins in each fraction were established by densitometry, as described in *Methods*. The relative abundance of each signaling molecule in the untreated cell, whole-cell extract was set to "1". The results are shown as mean values ± S.E. from 8–10 independent experiments. *Right-handed panel *displays the representative immunoblots, stained for Axin, β-catenin, GSK3β and p-Ser (9)-GSK3β.

Making use of the three independent strategies to suppress PP2A action, we probed further the linkage between PP2A and cellular content of key signaling elements in the Wnt3a-stimulated canonical pathway (fig. 3). OA, siRNA targeting PP2A C-subunit, or expression small *t *antigen were tested individually for their influence on the cellular content of β-catenin, Dvl2, Axin, GSK3β, phospho-GSK3β, PP2A C-subunit, and GAPDH (as a control). In order to facilitate comparisons, the cellular content of each molecule in the untreated cells was set as "1"; changes in cellular abundance in response to each of the three treatments were quantified and are reported as "fold-"changes. β-catenin levels, increasing 50% by OA treatment (fig. 1C), were similarly enhanced by either siRNA treatment or expression of small *t *antigen (fig. 3). The abundance of Axin as well as that of phospho-GSK3β increased 2- to 4-fold by suppression of PP2A action, the increases being somewhat greater in response to OA than to the other treatments. Cellular abundance of Dvl2 and GSK3β, like that of the control GAPDH, largely were unaffected by suppression of PP2A action. The abundance of PP2A was unaffected by either OA treatment or small t antigen expression. Treatment of siRNA resulted in ~70% suppression of PP2A C-subunit expression. Thus, the abundance of the scaffold Axin, of phospho-GSK3β, and of β-catenin itself are subject to regulation by PP2A, *i.e*., suppressing PP2A action increases their cellular content while enhancing Wnt3a stimulation of the canonical pathway (figs. 1, 3).

### Wnt3a action on cellular content of signaling elements is opposed by PP2A

**Figure 4:**
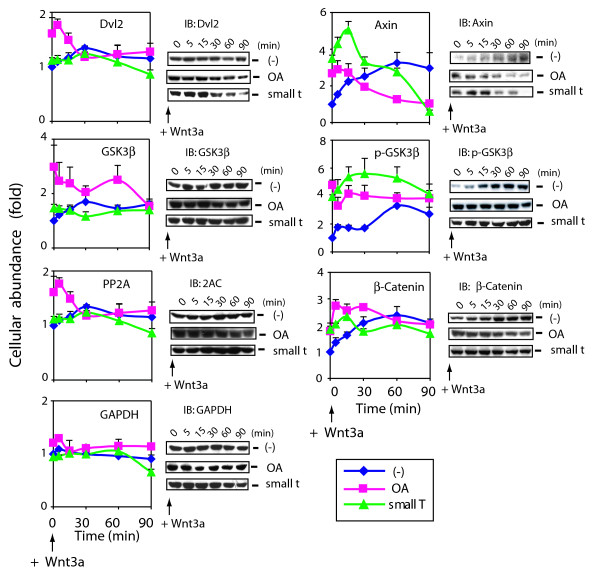
**Effects of inhibition of PP2A on cellular abundance of Wnt/β-catenin signaling elements**. Graphs display the relative cellular abundance of Wnt/β-catenin signaling elements in cells following stimulation with Wnt3a. F9 cells expressing Rfz1 were untreated or treated with Wnt3a in the presence or absence of OA for 0 to 90 min. For OA treatment, F9 cells expressing Rfz1 were treated with OA for 1 hr prior to Wnt3a stimulation. For small *t *antigen experiments, F9 cells were transiently co-transfected with Rfz1 and small *t *antigen, and then stimulated with Wnt3a for the indicated times. Cells were collected and lysed. Lysates (60–100 μg protein) were subjected to SDS-PAGE and analyzed by immunoblotting, blots stained with antibodies targeting the signaling molecules indicated. Bands were quantified by densitometry, as described in *Experimental Procedure *and values are displayed as "fold", with time = 0 set as "1". The results are shown as mean values ± S.E. from 6–8 independent experiments. Representative blots are displayed, as is the quantitative analysis of cellular content extracted from 6–8 separate experiments (graphs).

We investigated if PP2A also regulated Wnt3a-induced changes in cellular abundance of Dvl2, Axin, GSK3β, phospho-GSK3β, β-catenin, and GAPDH (as a control, fig. 4). Although having little influence on the abundance of Dvl2, GSK3β, PP2A, and the control GAPDH, Wnt3a stimulated a progressive increase in the cellular content of β-catenin (2-fold), phospho-GSK3β (2–3-fold), and Axin (3-fold) over 90 min. OA treatment as well as expression of small *t *antigen mimicked the effects of Wnt3a, increasing the expression of Axin (3- to 6-fold), phospho-GSK3β (4- to 5-fold) and β-catenin (2- to 3-fold). Thus, suppression of PP2A by any one of the strategies alone appears to mimic Wnt3a action, stabilizing β-catenin and phospho-GSK3β levels, while transiently increasing, then decreasing the cellular content of Axin and Dvl2 (fig. 4). PP2A levels increase modestly in response to either Wnt3a or suppression of PP2A. These data show that cellular content of Axin, phospho-GSK3β and β-catenin each reflect an inhibitory control of PP2A which can be reversed by suppression of PP2A action, thereby mimicking aspects of Wnt3a action.

### PP2A action & trafficking of Axin, Dvl2, and β-catenin

**Figure 5:**
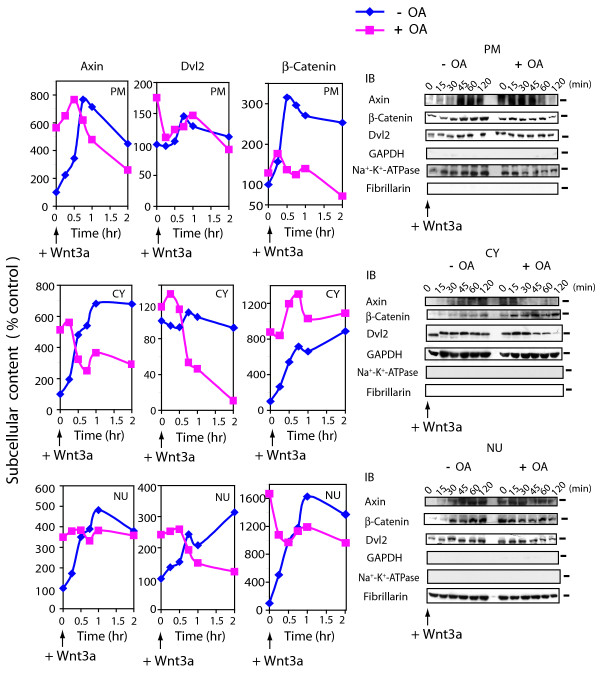
**Abundance of Axin, Dvl2 and β-catenin in response to Wnt3a: effects of okadaic acid**. F9 cells expressing Rfz1 were pretreated with or without 40 nM OA for 1 hr and then stimulated with Wnt3a in the presence or absence of OA for the indicated time periods. Cells were harvested and fractionated to subcellular fractions highly enriched in plasma membrane (PM), cytoplasm (CY), or nuclei (NU), as described in the *Methods*. Each fraction was separated by SDS-PAGE and the resolved protein transferred to blots stained with anti-Axin, anti-Dvl2 or anti-β-catenin antibodies. The enrichment of the subcellular fractions was established by staining immunoblots with antibodies to well known protein markers for PM, CY, and NU subcellular fractions. *Left-handed panel *displays the quantitative analysis of the blots for Wnt3a alone (blue line) or Wnt3a in the presence of OA (pink line). The data are displayed as the mean values "% of control" (*i.e*., t = 0 then set to 100%). *Right-handed panel *displays immunoblots. The results shown are derived from a single experiment, representative of two additional experiments.

We investigated the effects of the PP2A suppression on the intracellular shuttling of these Wnt/β-catenin pathway signaling molecules. OA-treated cells were stimulated with Wnt3a for up to 2 hr and the cells disrupted and then subjected to subcellular fractionation. Plasma membrane- (PM), cytoplasm- (CY), and nuclei- (NU) enriched subcellular fractions were prepared and the distributions of Axin, Dvl2, and β-catenin quantified (fig. 5). Most notable, OA alone mimics with high fidelity the effects of Wnt3a on the trafficking of Axin, Dvl2, and β-catenin. In response to Wnt3a, Axin initially traffics to PM, CY and NU fractions (fueled by its increase in cellular abundance) and OA alone mimics each of these effects of Wnt3a. OA treatment stimulated an initial rise in the amount of Dvl2 localized to PM and NU subcellular fractions. β-catenin levels increase markedly in CY and NU subcellular fractions in responseβ to OA.

In combination with Wnt3a stimulation, OA stimulated a substantial loss of NU and especially CY subcellular levels of Axin and of Dvl2 (fig. 5). CY levels of Axin fall several fold, while Dvl2 levels plummet to less that 15% within 2 hr of treatment with Wnt3a in combination with OA. For cells expressing the small *t *antigen (see additional file: Supplementary fig. 1) or those treated with siRNA targeting PP2A C-subunit for 30 hr (data not shown), Wnt3a stimulation provoked a similar decrease in CY content of Axin as well as Dvl2. OA in combination with Wnt3a provoked increased and sustained accumulation of β-catenin in CY and a markedly lesser accumulation in the PM and NU subcellular fractions (fig. 5). Expression of small *t *antigen yielded very similar data to that obtained with OA (see additional file: Supplementary fig. 1).

### GSK3β trafficking in response to Wnt3a

**Figure 6:**
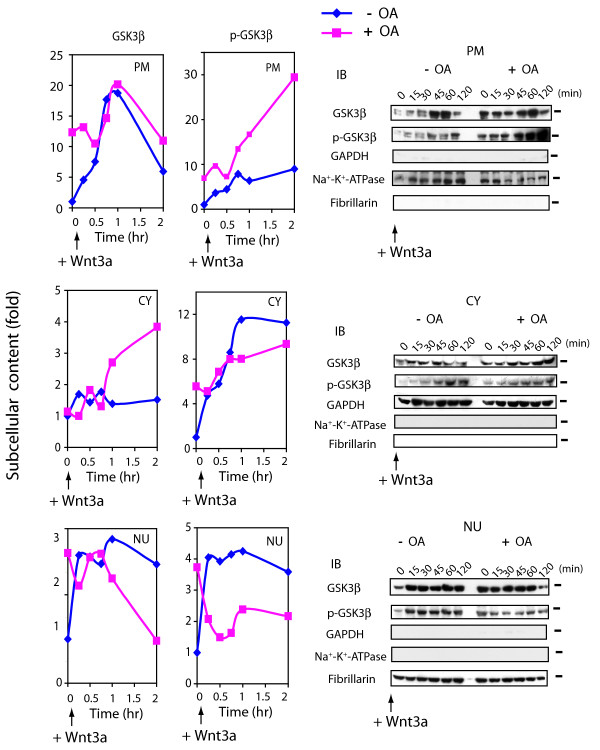
**Suppression of PP2A provokes shuttling and phosphorylation of GSK3β**. F9 cells expressed Rfz1 were treated without (-) or with (+) OA for 1 hr and then stimulated without (time + 0) or with Wnt3a for the indicated time periods. At indicated time points, cell cultures were harvested, disrupted, and subjected to subcellular fractionation to plasma membrane (PM), cytoplasm (CY) and nuclei (NU) fractions, as described in the *Methods*. Samples of each fraction were subjected to protein determination, SDS-PAGE, and the resolved proteins blotted and stained with antibodies specific for GSK3β and with antibodies specific for Ser9 phospho-GSK3β. *Left-handed panel *shows the quantitative analysis of the blots for Wnt3a alone (blue line) or Wnt3a in the presence of OA (pink line). The data are displayed as fold of control (time zero set to 1). *Right-handed panel *displays immunoblots stained with GSK3β and phospho-GSK3β (Ser 9) antibodies as well as antibodies specific for subcellular fractions, (*i.e*., marker proteins). The results shown are derived from a single experiment, representative of two additional experiments.

The activity of GSK3β becomes inhibited when the N-terminal serine residue (Ser 9) of the enzyme is phosphorylated, an event catalyzed by members of the AGC family of protein kinases [25,26]. Phospho-GSK3β is a substrate for PP2A, dephosphorylation of GSK3β by PP2A restores enzymatic activity [10]. OA provoked accumulation of GSK3β and phospho-GSK3β in the PM and the NU subcellular fractions, mimicking Wnt3a action (fig. 6). In the NU subcellular fraction, content of GSK3β as well as phospho-GSK3β increased 3- and 4-fold, respectively, in response to OA alone. For cells in which PP2A was suppressed by siRNA, nuclear GSK3β accumulated more than 5-fold and phospho-GSK3β by up to 2-fold (see additional file: Supplementary fig. 2A).

Most striking, Wnt3a stimulation in combination with OA treatment (or siRNA treatment or small *t *antigen expression) reverses the nuclear accumulation of both GSK3β and phospho-GSK3β, provoking a rapid loss of nuclear GSK3β with a sustained gain in the cytosol and a transient increase at the plasma membrane (fig. 6 and see additional file 2: Supplementary fig. 2B). Thus, PP2A activity appears to be essential for Wnt3a-induced nuclear retention of GSK3β. Loss of PP2A activity coupled with stimulation by Wnt3a leads to accumulation of GSK3β (especially phospho-GSK3β) in the PM and thereafter CY subcellular fractions, occurring at the expense of the NU subcellular fraction.

### Intracellular trafficking of PP2A is regulated by Wnt3a

**Figure 7:**
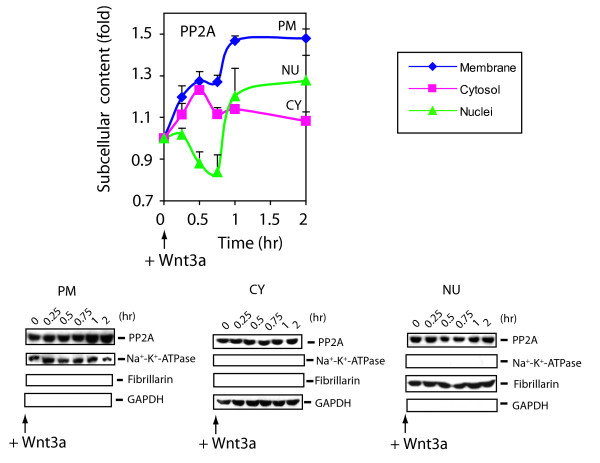
**PP2A shuttles to the plasma membrane and nuclear subcellular fractions in response to Wnt3a**. Cells expressing Rfz1 receptor were harvested at indicated time point after Wnt3a stimulation. Cells were fractionated to the plasma membrane (PM), cytoplasm (CY) and nuclei (NU), as described in the *Methods*. Subcelluar fractions obtained from control (time = 0) and Wnt3a-stimulated cells were analyzed by immunoblotting with anti-PP2A C-subunit antibody. PM (blue line), CY (pink line) and NU (green line) fractions are displayed. Stained protein bands were quantified and the values are presented as "fold of zero time point". Bottom *three panels *of immunoblots are stained with antibodies against the following subcellular fraction marker proteins: Na^+^-K^+^-ATPase (plasma membrane), GAPDH (cytoplasm) and fibrillarin (nuclei), respectively. The results are shown as mean values ± S.E. from 5 or more independent experiments. Representative blots are displayed, as is the quantitative analysis of cellular content extracted from a compilation of data from the separate experiments (graphs).

We wondered if PP2A itself was subject to trafficking in response to Wnt3a. Cells were treated without and with Wnt3a and then subjected to lysis and subcellular fractionation. Wnt3a stimulated rapid trafficking of and accumulation of PP2A in the PM and to a lesser extent in CY subcellular fractions, at the expense of the NU fraction (fig. 7). At 60 min post stimulation with Wnt3a, PP2A reverses course and begins to accumulate in the nucleus [much like Axin, β-catenin, and Dvl2 (fig. 5) and see additional file: Supplementary fig. 3A and 3B)]. PP2A localizes predominantly (>70%) in the cytosol; ~20% residing in the plasma membrane-enriched fraction, and the remaining <8%in the nuclear fraction of unstimulated F9 cells [20].

In unstimulated F9 cells, Dvl2 was observed to distribute in a similar manner, 80% in the cytosol, 10–15% in the plasma membrane-enriched fraction, and the balance (~5%) in the nucleus [20]. For Wnt3a-treated cells, trafficking of PP2A and Dvl2 were similar in magnitude and sign, both showing modest increases in plasma membrane- and nuclear-associated distribution (compare figs. 5, 7). In the presence of OA (or expression of small *t *antigen), Wnt3a stimulates a time-dependent loss of Dvl2 (fig. 5) and of PP2A (data not shown) in both the CY- and PM-enriched subcellular fractions. In the nuclear fraction, the accumulation of Dvl2 (fig. 5) and PP2A (data not shown) increase for 30 min. Thereafter, PP2A accumulates in the NU, whereas nuclear Dvl2 content declines (data not shown).

### PP2A directly associates with and dephosphorylates Dvl2

**Figure 8:**
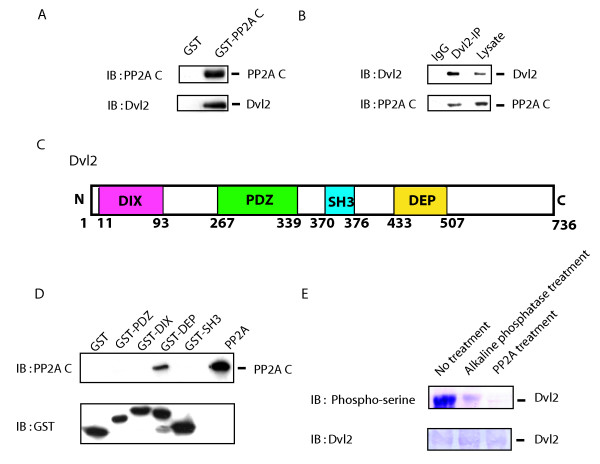
**PP2A associates directly with Dvl2 and dephosphorylates Dvl2**. *Panel A*, probing interaction of Dvl2 and PP2A *in vivo*. Whole-cell extracts (2 mg) prepared from F9 cells expressing Rfz1 were incubated with immobilized GST gel alone or with GST-PP2A C-subunit-immobilized gel. Bound proteins were released and resolved by SDS-PAGE. The resolved proteins were subjected to blotting and stained with either with anti-PP2A C subunit antibodies (*top panel*) or anti-Dvl2 antibodies (*bottom panel*). *Panel B*, pull-downs of Dvl2 from F9 cells reveal associated PP2A. F9 cells lysates were subjected to analysis by pull-downs of either Dvl2 or of mouse IgG (as a control). The immune precipitates were subjected to SDS-PAGE, immunoblotting, and staining with either anti-Dvl2 antibodies (top panel) or anti-PP2A C-subunit antibodies (bottom panel). *Panel C*, Dvl2 domains structure. *Panel D*, direct association of Dvl2 and PP2A *in vitro*. Purified mouse PP2A enzyme (PP2A, AC subunit dimers) was incubated with one of four domains of Dvl2 engineered as a fusion protein with GST and then immobilized: immobilized GST-PDZ, GST-DIX, GST-DEP, GST-SH3 and GST itself (as a control) at 4°C for 1 hr. The bound proteins were separated by SDS-PAGE, blotted, and stained with either anti-PP2A C-subunit antibodies or anti-GST antibodies. *Panel E*, PP2A dephosphorylates phospho-rDvl2. 6-Histidinyl-tagged phosphorylated Dvl2 was expressed in Sf9 cell and purified by Ni-NTA column chromatography as described in *Methods*. Purified phospho-rDvl2 protein was incubated with either purified calf alkaline phosphatase or with purified PP2A for 1.5 hr. The incubation was terminated by addition of SDS-PAGE sample buffer. The samples were subjected to SDS-PAGE, blotted, and stained with either anti-phospho-Ser antibodies (*top of panel*) or with anti-Dvl2 antibodies (*bottom of panel*). The immune complexes were made visible by use of an alkaline phosphatase-conjugated, second antibody and BCIP/NBT as substrates. The results shown are from a single experiment, duplicated with essentially similar results.

The ability of PP2A inhibition to influence the Wnt/β-catenin pathway and the trafficking of PP2A in response to Wnt3a stimulated us to hypothesize that this phosphoprotein phosphatase may interact directly with Dvl2. PP2A could well associate with Dvl2 directly or indirectly via Axin, APC, or some other unidentified protein. To test the hypothesis that PP2A binds directly to Dvl2, whole-cell lysates were subjected to pull-downs with either a fusion protein of PP2A C-subunit with glutathione-S-transferase (GST) immobilized to glutathione gel beads or GST itself immobilized to gel beads, as a control (fig. 8A). Pull-downs with PP2A C-subunit-GST beads, but not GST beads alone, reveal bound Dvl2, as evidenced by immunoblotting of the pull-down complexes. Pull-downs targeting the Dvl2 rather than PP2A were conducted and the immunoblotting confirmed the presence of PP2A in the Dvl2-based pull-downs (fig. 8B).

The possibility that association of Dvl2 and PP2A is not direct, but rather indirect via other interacting protein(s) such as Axin, APC, *etc*. required further analysis. To obviate this criticism and to determine whether the association of Dvl2 with PP2A is direct, we made use of GST-fusion protein with defined fragments of Dvl2 that contain one of the four major domains, *e.g*., DIX, PDZ, DEP, and an SH3 binding domain (fig. 8C). The proline-rich putative SH3 binding domain of Dvl2 is located C-terminal to the PDZ domain, but N-terminal to the DEP domain [27]. Purified PP2A was incubated *in vitro *with individual GST-fusion domains, GST-PDZ, GST-DIX, GST-DEP, or GST-SH3 as well as the GST immobilized to the glutathione bead itself (as a control). Pull-downs of the GST-tagged fusion molecules establish PP2A binding to the GST-fusion protein harboring the DEP domain of Dvl2, but no other domain (fig. 8D). Each of the other Dvl2 domains (*i.e*., DIX-, PDZ- and SH3-) as well as the GST immobilized control failed to display binding of purified PP2A. These observations demonstrate that Dvl2 is capable of binding PP2A directly, interacting through the DEP domain found in the C-terminal third of the Dvl.

Dsh/Dvls are well-known phosphoproteins [28]. We show the Dvl2 binds PP2A both *in vitro *using purified components in a reconstituted system as well as in assays making use of whole-cell lysates. These observations call the question if phospho-Dvl2 indeed is a substrate for PP2A? To investigate this possibility, we assayed the ability of PP2A to dephosphorylate phospho-Dvl2 directly *in vitro *(fig. 8E). Histidine-tagged mouse Dvl2 was expressed in Sf9 insect cells using a baculovirus-induced expression system. The rDvl2 expressed in the insect cells was purified by Ni-NTA column chromatography and found to be highly phosphorylated (fig. 8E, left-lane). The purified phospho-rDvl2 was treated either without (no treatment), or with purified calf alkaline phosphatase, or with purified PP2A. The phosphorylation status of phospho-Dvl2 was established readily by SDS-PAGE, blotting, and staining of the treated rDvl with phospho-Ser-specific antibodies (fig. 8E). Treatment with purified PP2A was found to effectively dephosphorylate phospho-rDvl2 *in vitro*. Treatment with purified alkaline phosphatase also dephosphorylates purified, phospho-Dvl2. These results demonstrated that Dvl2 indeed is as a substrate for PP2A.

### Wnt3a stimulated PP2A binding to Dvl2

**Figure 9:**
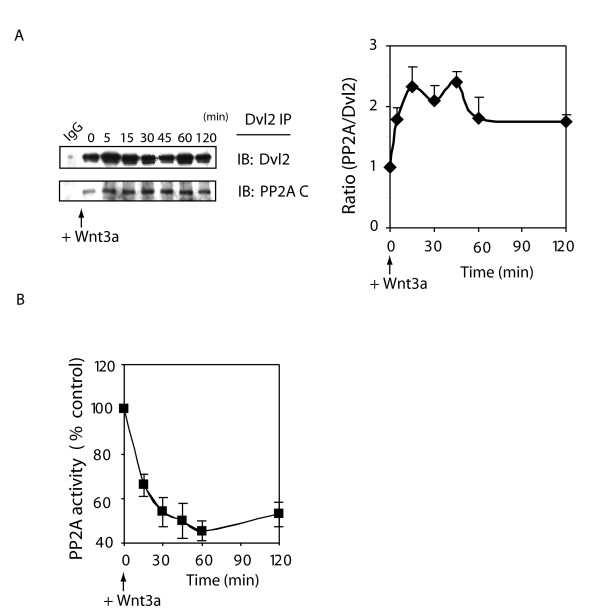
**Dvl2 interacts with PP2A and PP2A activity is attenuated in response to Wnt3a**. *Panel A*, Association of Dvl2 and PP2A was investigated in the cytosolic fraction. Cell lysates (1 mg) were immunoprecipitated with Anti-Dvl2 antibody. Bound proteins were resolved by SDS-PAGE, immunoblotting, and made visible by staining with either anti-Dvl2 or anti-PP2A C-subunit antibodies. Dvl2 and PP2AC association is displayed as "fold" (time = 0, set to "1"). Representative blots are displayed. The results are shown as mean values ± S.E. from 5 independent experiments.* Panel B*, F9 cells expressing Rfz1 were stimulated with purified Wnt3a for the indicated time. Cells were washed with PBS and lysed. Cell lysates were applied to a small Sephadex G-50 column to remove small molecular substances that interfere with the assay, and PP2A activity determined. Results were shown as the mean values ± S.E. from 8 independent experiments.

Dvl2 is a phosphoprotein, transiently phosphorylated in cells in response to stimulation by Wnt3a [28]. The demonstration of a direct association of Dvl2 with PP2A (via DEP domain, fig. 8) provokes the question if Wnt3a stimulates protein-protein interactions between PP2A and Dvl2. Association of Dvl2 and PP2A was assayed in pull-downs employing anti-Dvl2 antibody, followed by immunoblotting of the pull downs with anti-Dvl2 and anti-PP2A C subunit antibodies. PP2A binding to Dvl2 increased in response to stimulation by Wnt3a and sustained over 2 hr (fig. 9A).

We also tested if the activity of PP2A was influenced by stimulating cells with Wnt3a (0–2 hr). The phosphoprotein phosphatase activity of PP2A was probed in cells stimulated with Wnt3a. PP2A "activity" is defined as that PPTase activity sensitive to inhibition by OA. Wnt3a stimulation of the cells provoked a sharp decline in PP2A activity (fig. 9B). The Wnt3a-induced loss of PP2A was ~35% within 15 min of stimulation, reaching 50% loss within 30 min and remained reduced for up to 2 hr post stimulation with Wnt3a (fig. 9B). By 3 hrs post Wnt3a-stimulation, PP2A activity returned to normal levels (data not shown). Thus, Wnt3a stimulation provokes trafficking of PP2A and Dvl2, binding of PP2A to Dvl2, and attenuation of PP2A enzymatic activity.

## Discussion

The goal of the current study was to probe the role of PP2A action in the signaling of the Wnt canonical pathway, focusing upon the role of PP2A in the regulating the signaling, the abundance and trafficking of key molecules in this Wnt3a/β-catenin response culminating in the activation of Lef/Tcf-sensitive transcription. Since the chemical inhibition of PP2A by okadaic acid is selective, but not specific, we employed two additional means to suppress PP2A activity, *i.e*., targeted suppression of the C-subunit of PP2A with siRNA and expression of the small *t *antigen, which binds to and inhibits PP2A activity [23,24,29]. Although the data gained from the three independent strategies were not identical in all read-outs, for the most part the results of show that PP2A regulates the Wnt-canonical pathway signaling at several key points of regulation, *e.g*., cellular abundance, trafficking, and nuclear retention of key signaling elements, Dvl2, Axin, GSK3β, and β-catenin itself.

Independent of the means of suppressing PP2A activity, there was a corresponding increase in the accumulation of the less active, phospho-GSK3β which mimicked the effects of Wnt3a. All three approaches employed to suppress PP2A provoked the accumulation of phospho-GSK3β, potentiated activation of the Lef/Tcf-sensitive transcriptional response to Wnt3a. Thus, the suppression of PP2A activity mimics Wnt3a in the absence of the ligand, while potentiating yet not mimicking the ability of Wnt3a to stimulate the Lef/Tcf-sensitive transcriptional response.

PP2A action in F9 cells includes effects on the cellular abundance of signaling elements in the Wnt canonical pathway. The increased cellular content of phospho-GSK3β, Axin, and β-catenin in response to OA provides at least a partial basis for the Wnt-mimetic effects of PP2A inhibition. Further support for this observation was garnered in parallel studies performed in cells in which the cellular expression of PP2A was suppressed by use of siRNAs or by the expression of the PP2A inhibitor SV40 small *t *antigen. Taken together, these studies highlight the necessity to quantify cellular abundance of individual signaling elements in the Wnt canonical pathway, as the cellular abundance is dynamic and the changes can be quite significant (*e.g*., OA-stimulated a ~4-fold change in the cellular content of Axin and phospho-GSK3β).

The influence of PP2A on Wnt canonical signaling pathway was not restricted to regulating the cellular content of several key signaling molecules in the pathway, but also included trafficking of these signaling molecules among the plasma membrane-, cytosol-, and nuclear-enriched subcellular fractions. With respect to the trafficking of Dvl2, Axin, phospho-GSK3β, and β-catenin, chemical inhibition of PP2A (in the absence of Wnt3a) was observed to increase the trafficking of each to both the plasma membrane and nuclear subcellular fractions, much like Wnt3a stimulation. Suppression of PP2A activity by expression of small *t *antigen provoked a very similar effect, providing compelling evidence that PP2A negatively regulates the Wnt canonical pathway and any means employed to suppress PP2A action is Wnt-mimetic with respect to some basic regulation of cellular abundance and trafficking of signaling molecules.

Quite unexpected and separate from these effects of PP2A on cellular content and trafficking of signaling molecules is that on the retention of signaling molecules in subcellular fractions, *e.g*., cytosol and nucleus, when PP2A action is suppressed and the cells stimulated with Wnt3a. Although OA alone is Wnt-mimetic with regard to cytosolic accumulation of Dvl2, Wnt3a stimulation in combination with OA provoked a loss of Dvl2 cellular content, with obvious loss in the cytosol as well as the nucleus. Similarly, OA and Wnt3a in combination stimulated at dramatic loss in plasma membrane-associated and nuclear β-catenin. Thus, nuclear accumulation of β-catenin *per se *does not translate into activation of Lef/Tcf-sensitive transcription when PP2A activity is suppressed. A more detailed understanding of phosphorylation and β-catenin regulation of Lef/Tcf-sensitive genes will require additional studies, but such observations would likely have escaped notice in the absence of quantifiable biochemical data (current work).

Finally, we report that Dvl2 binds PP2A in response to Wnt3a stimulation. The interaction between these two key molecules in the Wnt canonical pathway is direct, as established in an *in vitro *reconstituted system. Furthermore the binding site for PP2A can be ascribed to a region of Dvl2 that contains the well-known DEP domain. The binding of PP2A to Dvl2 is shown to "silence" or attenuate the activity of PP2A. At present mechanism of inhibition of PP2A is not known. The DEP domain of Dishevelled has been shown to be required for function of the Wnt canonical pathway, *i.e*., expression of Dishevelleds with mutations of the DEP domain inhibits Wnt activation of Lef/Tcf-sensitive transcription [30] and expression of the DEP domain alone (acting as a dominant negative) similarly blocks the trafficking of Dishevelled as well as the activation of the canonical pathway in response to Wnt [31]. The DEP domain of Dvl2 and/or its N-terminus flanking sequences has been shown to be essential to Dvl2-mediated signaling [32], providing additional support for our observations here.

## Conclusion

Wnt3a stimulation may well provoke an inhibition of GSK3β by preserving the phosphorylated, inhibited form of GSK3β from dephosphorylation catalyzed by PP2A as well as catalyzing the binding to Dvl2 that enables trafficking. It is clear that the ability of PP2A to dampen the Wnt canonical pathway includes effects at several discrete levels: protein expression, protein trafficking; retention of molecules in subcellular compartments (especially the nucleus), and regulation of enzymatic activity of several key players. The ability of PP2A to bind to several key signaling molecules in the pathway (*e.g*., Axin, APC gene product, and now Dvl2), regulate cellular expression of some and trafficking of others, may well explain the ability of PP2A suppression to mimic many actions of Wnt3a, but fail to activate the Lef/Tcf-sensitive transcriptional response. Trafficking of signaling elements such as β-catenin in response to Wnt3a may require suppression of PP2A, whereas "activation" of nuclear β-catenin enabling its stimulation of the Lef/Tcf-sensitive transcriptional response may be likewise PP2A-dependent and similarly blocked by PP2A inhibitors.

## Methods

### Materials

The following reagents were purchased from the indicated commercial supplier(s): anti-Dvl2 and SV40 anti-small *t *antigen antibodies were from Santa Cruz Biotechnology (Santa Cruz, CA); anti-GSK3β antibody from Cell Signaling (Danvers, MA); anti-Axin, anti-PP2A C subunit, and anti-phosphoserine-9-GSK3β antibodies from Upstate Biotechnology (Lake Placid, NY); anti-β-catenin antibody and okadaic acid (OA) from Sigma (St. Louis, MO); anti-glyceraldehyde-3-phosphate dehydrogenase (GAPDH), anti-Na^+^-K^+^-ATPase, and anti-fibrillarin antibodies from Abcam (Cambridge, MA); polyclonal anti-phospho-serine antibody from Zymed Laboratories (South San Francisco, CA); a second anti-Axin antibody from R&D systems (Minneapolis, MN); Ni-NTA resin from Qiagen (Valencia, CA); glutathione-coupled agarose from Molecular Probes (Eugene, OR); calf intestinal alkaline phosphatase, pFastBacHTb, LipoFectamine 2000, and Bac-to-Bac baculovirus system from Invitrogen (Carlsbad, CA); siRNAs targeting PP2A C-subunit expression and a scrambled sequence control siRNA were purchased from Ambion (Austin, TX); Immobilon membrane from Millipore (Bedford, MA) ;and, Wnt3a from R& D Systems (Minneapolis, MN). The plasmid expression vector harboring the SV40 small *t *antigen was a kind gift of Dr. Bryce M. Paschal (University of Virginia).

### Cell culture and transfection

The mouse F9 teratocarcinoma cells (F9) were obtained from ATCC collection (Manassas, VA) and maintained in Dulbecco's modified Eagle's medium (DMEM) supplemented with 15% fetal bovine serum and penicillin/streptomycin at 37°C in 5% CO_2_. Cells were transiently transfected with an expression vector (pCDNA3) harboring the rat Frizzled-1 (Rfz1) using lipofectamine (Invitrogen) according to the manufacture instructions. For some experiments, cells were co-transfected with Rfz1 and SV40 small *t *antigen. Cells were stimulated with purified Wnt3a at a final concentration of 20 ng/ml. For okadaic acid treatment, cells were pretreated with 40 nM OA for 1 hr and then stimulated without and with Wnt3a, in the continued presence of OA.

### Lef/Tcf-sensitive transcriptional reporter gene assay

F9 cells were grown on 12-well plates and co-transfected with Rfz1 and Super8xTOPFlash (M50) in presence or absence of small t antigen [33]. Cells were stimulated with Wnt3a for up to 8 hr after 1–2 days transfection and then washed with phosphate-buffered saline (PBS) two times. Cell lysates were collected in a lysis buffer [12.5 mM Tris-H_3_PO_4 _pH 7.8, 1 mM Trans-1, 2-cyclohexanediaminetetraacetic acid (CDTA), 2 mM DTT, 10% glycerol and 1% Triton X-100 (Promega, Madison, WI)]. Luciferase activity was determined according to the manufacture's instructions (Stratagene, La Jolla, CA). For knockdown experiments, the siRNAs (either targeting the PP2A C-subunit or a scrambled sequence "control" from Ambion) were introduced into F9 cells using Lipofectamine 2000 one day prior to the co-transfection with Super8xTOPFlash (M50) and Rfz1. For indicated experiments, cells were treated with 30 nM OA for 1 hr and then stimulated with Wnt3a in the presence of OA.

### Disruption of cells and subcellular fractionation

Cells were untreated or stimulated with purified Wnt3a for the times indicated. Cells were washed with PBS twice and harvested. Cells underwent disruption and subcellular fractionation as described previously [20,34]. Cells were disrupted by passage through a 23-gauge needle 10 times in buffer A (10 mM Hepes, pH 7.0, 5 mM MgCl_2_, 25 mM KCl, 1 mM Na_3_VO_4_, 50 mM NaF, 1 mM PMSF, 10 μg/ml leupeptin, and 10 μg/ml aprotinin) and were immediately mixed with an equal volume of buffer A containing of 0.25 M sucrose. Nuclei and unbroken cells were collected by centrifugation at 500 × *g *for 10 min. These pellets were used for the preparation of nuclear-enriched subcellular fraction. Pellets were lysed in a buffer (10 mM Hepes, pH 7.0, 0.5 M KCl, 1.5 mM MgCl_2_, 0.2 mM EDTA, 1 mM DTT, 1 mM PMSF, 10 μg/ml leupeptin, and 10 μg/ml aprotinin). This fraction represents the standard subcellular, nuclear-enriched fraction. After nuclei (and any unbroken cells) were removed, EDTA was added to a final concentration of 10 mM, followed by centrifugation at 16,000 × *g *for 15 min. The supernatant fractions then were centrifuged at 100,000 × *g *for 1 hr. The 100,000 × *g *supernatant represents the standard subcellular, cytosol-enriched fraction. The 100,000 × *g *pellets were lysed in a buffer (10 mM Tris-HCl, pH 7.5, 5 mM EDTA, 150 mM NaCl, 2 mM Na_3_VO_4_, 0.5% NP-40 and 1% Triton). This fraction represents the standard subcellular, plasma membrane-enriched fraction. Protein concentration was determined by the Bradford assay. The purity of these enriched subcellular fractions was established by subjecting aliquots to SDS-PAGE, immunoblotting, and staining with the following marker proteins: anti-Na^+^-K^+^-ATPase (plasma membrane), anti-GAPDH (cytoplasm), and anti-fibrillarin (nucleus) antibodies.

### Immunoblotting and quantification analysis of proteins

Proteins (60–100 μg samples) were analyzed by SDS-polyacrylamide gel electrophoresis and transferred to Immobilon membrane (Millipore, Bedford, MA). The blots were stained with the indicated antibodies. Immunoreactive bands were detected using a horseradish peroxidase-conjugated, secondary antibody in tandem with ECL chemiluminescence. Exposed films were scanned by calibrated Umax 1000 absorbance scanner equipped with SilverFastAi software (LaserSoft Imaging Inc. Longboat Key, FL). The bands were quantified by use of Aida software (Raytest, Germany). For Axin analysis, two distinct epitope antibodies were applied.

### Knockdown of PP2A C-subunit by siRNA

siRNA sequences designed to suppress the expression of PP2A C subunit are as follows: GGAUAUUACUCUGUUGAAAtt and UUUCAACAGAGUAAUAUCCtc. Treatment with these siRNA effectively suppressed the expression of PP2A C-subunit. After treatment with PP2A C siRNA for 24 hr, F9 cells were transfected with M50 and an expression vector harboring the Rfz1. On the following day, F9 cells were stimulated with or without Wnt3a for 2–10 hr. For the subcellular fractionation experiments, F9 cells were grown on 100 mm culture dishes and siRNAs were introduced to cultures one day prior transfection with the expression vector harboring Rfz1. Cells were cultured one additional day and stimulated with Wnt3a for 0–2 hr. A scrambled sequence siRNA designed by Ambion was used as a "control".

### Expression and purification of Dvl2 in Sf9 cells

The pFastBac HTb (Invitrogen) vector was exploited to create a fusion protein with a 6-histidinyl sequence at the N-terminus of protein. We reengineered this vector to enable dual tagging of the fusion molecule with both an N- and a C-terminal 6-histidinyl-tag, by modification of the multi-cloning site of the vector at the C-terminus. The full-length Dvl2 construct was generated by PCR. The 5' PCR primer had the sequence CGGGATCCATGGCGGGCAGCAGC, and the 3' primer was CGGAATTCGCATAACATCCACAAAAAACTC. These primers had 23 nucleotides (5' primer) and 30 nucleotides (3' primer) of complementarity with the template and encode unique restriction sites (*BamHI *at the 5'-end and *EcoRI *at the 3'-end). PCR product was ligated into plasmid N- and C-terminal dually histidinyl-tagged pFastBacHTb vector. Dvl2 was expressed in *Spodoptera frugiperda *(Sf9) cells using Bac-to-Bac Baculovirus system (Invitrogen). For protein expression, Sf9 cells were infected with recombinant Dvl2 baculovirus at a multiplicity of infection of 3.0. After 3 days infection, Sf9 cells were harvested and washed with phosphate-buffered saline twice. Cells were lysed by use of a French pressure cell two times in 20 mM Tris-HCl buffer (pH 8.0) containing 1% deoxycholate, 2 mM Na_3_VO_4_, 20 mM NaF, 5 mM 2-mercaptoethanol, 10 μg/ml leupeptin, 10 μg/ml aprotinin and 1 mM phenylmethylsulfonyl fluoride. Lysates were centrifuged at 40,000 × *g *for 30 min. The supernatant was filtered (0.45 μm), and diluted 4 times with a buffer lacking the deoxycholate and applied to a 3-ml of Ni-NTA column (Qiagen). The column was washed exhaustively with buffer containing of 20 mM imidazole, 0.5 M NaCl, 2 mM Na_3_VO_4_, 10% glycerol, 5 mM mercaptoethanol, 20 mM NaF, 20 mM Tris-HCl (pH 8.0). The rDvl2 retained on the washed affinity matrix was efficiently eluted with buffer containing of 100 mM imidazole, 2 mM Na_3_VO_4_, 10% glycerol, 5 mM mercaptoethanol, 20 mM Tris-HCl (pH 8.0). The protein product was concentrated by use of an Amicon Ultra-15 column (Millipore).

### GST-PP2A C-subunit pull-down experiments

GST fusion protein of PP2A C-subunit was immobilized on glutathione-derivatized agarose matrix. Cell extracts (2 mg) from Rfz1-expressing F9 cells were incubated with GST-PP2A C-subunit or GST alone for 1 hr. The gels were washed with phosphate-buffered saline containing of 0.5% of NP-40. The bound proteins were separated by SDS-PAGE and analyzed with anti-PP2A C-subunit and anti-Dvl2 antibodies.

### Dvl2 GST fusion proteins

GST-DIX (amino acids 11–93 of Dvl2), GST-PDZ (amino acids 267–339 of Dvl2), GST-SH3 (amino acids 370–376 of Dvl2), GST-DEP (amino acids 433–507 of Dvl2) and GST were expressed in *E. coli *BL21 cells as fusion proteins. The fusion proteins were purified using glutathione-agarose, as described previously [35].

### Pull-down experiments using GST fusion proteins

PP2A [purified PP2A (AC dimer), 0.2 μg, Upstate Biotechnology] was incubated for 1 hr at 4°C alone or in combination with GST-fusion proteins of the DIX, PDZ, DEP, or SH3 domains of Dvl2 that were derivatized to gels. The gels were washed with PBS containing of 0.5% of NP-40. The bound proteins were eluted by SDS-sample buffer and analyzed by SDS-PAGE. The resolved proteins were subjected to blotting and then stained with anti-GST and anti-PP2A C-subunit antibodies.

### PP2A activity assay

Rfz1-expressing F9 cells were untreated or stimulated with purified Wnt3a for the times indicated. Cells were washed with PBS twice and harvested. Cells were lysed in a buffer (10 mM Tris-HCl, pH 7.5, 5 mM EDTA, 150 mM NaCl, 2 mM Na_3_VO_4_, and 1% Triton, 1 mM PMSF, 10 μg/ml leupeptin, and 10 μg/ml aprotinin). Cell lysates were applied to a small Sephadex G-50 column to remove low molecular weight substances, which interfered PP2A assay. The cell lysates were aliquoted and stored at -80°C. Each aliquot was only thawed once for the PP2A activity assay. For PP2A activity, aliquot of the fractions were assayed using *p*-nitrophenyl phosphate (pNPP) as a substrate [36]. Reaction was carried out at 30°C in a mixture containing 10 mM Tris-HCl (pH.7.4), 10 mM MgCl_2_, 2 mM MnCl_2_, 1 mM DTT and 100 μg BSA in the presence or absence of 5 nM OA. The reaction was terminated by addition of 2.5 M Na_2_CO_3 _and the optimal density was measured at 405 nm. PP2A activity was defined as phosphoprotein phosphatase activity sensitive to inhibition by 5 nM OA.

## Abbreviations

APC : *adenomatous polyposis coli *gene;

C-subunit : conserved catalytic subunit;

DMEM : Dulbecco's modified Eagle's medium;

Dvl : Dishevelled;

Frizzled : Fz;

GAPDH : glyceraldehyde-3-phosphate dehydrogenase; 

GSK 3 : glycogen synthase kinase 3;

GST : glutathione S-transferase;

OA : okadaic acid;   

PP2A : protein phosphatase-2A;   

SDS-PAGE: sodium  dodecyl sulfate-polyacrylamide gel electrophoresis.

## Competing interests

The author(s) declare that they have no competing interests.

## Authors' contributions

NY designed the study, analyzed the data and drafted the manuscript. CCM participated in coordination and helped to draft the manuscript. All authors read and approved the final manuscript.
